# Rapid Central Visual Field Progression Rate in Eyes with Open-Angle Glaucoma and Choroidal Microvasculature Dropout

**DOI:** 10.1038/s41598-019-44942-5

**Published:** 2019-06-12

**Authors:** Youn Hye Jo, Junki Kwon, Daun Jeong, Kilhwan Shon, Michael S. Kook

**Affiliations:** Department of Ophthalmology, University of Ulsan, College of Medicine, Asan Medical Center, Seoul, Korea

**Keywords:** Optic nerve diseases, Risk factors, Prognostic markers

## Abstract

Central visual field (CVF) loss has been linked to poorer vision-related quality of life in eyes with open-angle glaucoma (OAG) and can occur even in early-stage OAG eyes. The present study investigated whether the detection of choroidal microvasculature dropout (CMvD) during follow-up, which may be a sign of perfusion deficiency in the optic nerve head, is associated with rapid rate of CVF loss in early-stage OAG eyes. This study included 44 Korean OAG eyes with CMvD, identified by optical coherence tomography angiography, and 44 Korean OAG eyes without CMvD matched for age and severity of visual field loss at initial presentation. The rates of mean threshold changes in global and CVF were compared in eyes with and without CMvD using a linear mixed model. Clinical variables associated with rapid rate of CVF progression were also identified using a linear mixed model. The CVF progression rate was significantly higher in eyes with CMvD than in those without CMvD (−0.584 dB/year vs. −0.190 dB/year; P < 0.001) and detection of CMvD during follow-up was an independent predictor of rapid CVF progression rate. The presence of CMvD may aid in identification of high-risk patients who may show faster CVF progression and require more aggressive treatment.

## Introduction

Choroidal microvasculature dropout (CMvD) within β-zone parapapillary atrophy (β-PPA), as detected by optical coherence tomography angiography (OCT-A), may represent choroidal vascular impairment within β-PPA adjacent to the optic nerve head (ONH)^1,2^ Because the parapapillary choroid is closely associated with ONH perfusion due to its proximity to and common blood supply from the short posterior ciliary (SPC) artery^3–5^, the presence of CMvD may indicate decreased perfusion of the ONH in glaucomatous eyes. Eyes with CMvD may therefore be associated with greater susceptibility to progressive glaucomatous damage owing to vascular insufficiency in the ONH and its surrounding structures. Recently, CMvD detected during follow-up has been associated with a higher probability of retinal nerve fiber layer (RNFL) thinning, especially in eyes with open-angle glaucoma (OAG) accompanied by disc hemorrhage (DH), or visual field (VF) progression^6,7^. Nevertheless, the clinical implications of CMvD remain elusive, particularly with respect to its prognostic role in glaucoma during follow-up.

The central visual field (CVF), which includes the 12 points in the central 10° VF area on standard 24-2 VF testing, is of paramount importance, as it is linked to vision-related quality of life (QOL) measured by the National Eye Institute Visual Function Questionnaire^8^. Patients with glaucoma assign the greatest importance to tasks that use the CVF, such as reading and driving^9,10^. Therefore, CVF preservation is a key concern in glaucoma management. Our group previously reported that CMvD is associated with CVF loss in glaucoma patients^11^, indicating that CMvD may have important clinical relevance to CVF preservation during glaucoma management.

Because CMvD may indicate perfusion deficiency in the ONH and is associated with CVF loss, we hypothesized that glaucomatous eyes with CMvD may be at greater risk of rapid CVF progression than glaucomatous eyes without CMvD. To our knowledge, however, it has not been determined whether the detection of CMvD is associated with a higher rate of VF loss at a specific location (i.e., the CVF region) in glaucoma patients. Thus, this study was designed to evaluate the relationship between the detection of CMvD during follow-up and the rate of CVF deterioration by comparing the rates of global and central VF progression in OAG eyes with and without CMvD. This study also analyzed the clinical variables associated with rapid CVF progression in patients with early-stage OAG.

## Results

Ninety-four consecutive OAG eyes of 79 patients that met the initial inclusion criteria were evaluated. Of these, six eyes (6.4%) from six patients (7.6%) were excluded because the quality of their OCT-A images was too poor for CMvD evaluation. Thus, 88 OAG eyes of 73 patients met our final inclusion criteria, including 44 OAG eyes with CMvD and 44 OAG eyes without CMvD matched for age and VF mean deviation (MD). Interobserver agreement regarding the determination of the presence of CMvD was excellent (k = 0.975, P < 0.001). The intraclass correlation coefficients (ICC) for measurements of β-PPA-to-optic disc area ratio (β-PPA/disc area) was 0.986 (95% confidence interval [CI], 0.977–0.991; P < 0.001).

The demographic and clinical characteristics of the two groups are summarized in Table 1. The two groups differed significantly in gender distribution, β-PPA/disc area ratio, incidence of baseline CVF defects and of VF progression based on GPA (guided progression analysis) during follow-up, and number of VF tests (P < 0.05 each). However, there were no significant between group differences in age; laterality; Axial length (AL); spherical equivalent (SE); central corneal thickness (CCT); number of topical eye medications; incidence of DH; baseline location of hemifield VF defects (superior vs. inferior); baseline and follow-up mean and peak intraocular pressure (IOP); IOP fluctuation (standard deviation [SD]) of IOP); follow-up period; baseline/last follow-up VF MD; and baseline pattern standard deviation (PSD) (P > 0.05 each). Furthermore, the presence of hypertension, diabetes mellitus, Raynaud symptoms, and migraine, as well as Systolic blood pressure (SBP), diastolic blood pressure (DBP), and Mean ocular perfusion pressure (MOPP), did not differ significantly in the two groups (P > 0.05 each).

**Table 1:** Comparison of clinical and demographic characteristics of eyes with open-angle glaucoma with (CMvD+) and without (CMvD−) CMvD.

Characteristics	CMvD(−), n = 44	CMvD(+), n = 44	P value
Age, years	54.43 ± 11.96	54.73 ± 13.21	0.913
Gender (male:female)^†^	24:20	14:30	0.031*
Laterality (Rt:Lt)^†^	20:24	18:26	0.667
Axial length, mm	24.18 ± 1.51	24.38 ± 1.16	0.623
SE, D	−1.00 ± 1.99	−1.56 ± 2.43	0.238
CCT, µm	533 ± 32.71	529.78 ± 33.86	0.666
Hypertension, %^†^	22.7	18.2	0.597
Diabetes mellitus, %^†^	11.4	6.8	0.458
Raynaud symptoms, %^†^	0 (0/30)	3 (3/33)	0.094
Migraine, %^†^	6.7 (2/30)	6.1 (2/33)	0.922
SBP, mmHg	127.46 ± 7.70	126.54 ± 12.21	0.743
DBP, mmHg	74.73 ± 7.84	73.89 ± 7.26	0.685
MOPP, mmHg	27.57 ± 23.58	29.52 ± 23.02	0.695
Eye-drops (n)	1.43 ± 0.82	1.50 ± 0.79	0.692
DH, %	25	29.5	0.632
β-PPA/disc ratio	1.53 ± 0.25	1.73 ± 0.32	0.001*
Baseline IOP, mmHg	15.65 ± 1.75	13.50 ± 1.23	0.196
Mean F/U IOP, mmHg	13.64 ± 1.75	13.50 ± 1.23	0.657
Peak F/U IOP, mmHg	16.77 ± 2.03	16.03 ± 1.97	0.334
F/U IOP fluctuation, mmHg	2.48 ± 6.27	1.56 ± 0.50	0.267
Follow-up, years	5.77 ± 1.54	5.77 ± 1.83	0.999
VF test number (n)	7.32 ± 1.31	8.05 ± 1.96	0.044*
Location of defect (sup:inf)	30:14	35:9	0.225
Type of defect (cent:pph)	8:36	28:16	<0.001*
VF progression, %	27.3	56.8	0.005*
**Visual field**			
Baseline MD, dB	−2.08 ± 1.75	−2.48 ± 1.69	0.278
Baseline PSD, dB	4.01 ± 2.76	5.30 ± 3.33	0.052
Last F/U MD, dB	−3.36 ± 2.62	−4.37 ± 2.26	0.055

Data are reported as mean ± standard deviation or n (%). *P < 0.05 by independent t-tests unless otherwise indicated. ^†^Chi-squared tests.

Abbreviations: CMvD, choroidal microvasculature dropout; n, number; Rt, right; Lt, left; SE, spherical equivalent; D, diopter; SBP, systolic blood pressure; DBP, diastolic blood pressure; MOPP, mean ocular perfusion pressure; DH, disc hemorrhage; n, number; β-PPA, β-zone parapapillary atrophy; IOP, intraocular pressure; F/U, follow-up; CCT, central corneal thickness; VF, visual field; MD, mean deviation; dB, decibel; PSD, pattern standard deviation; sup, superior; inf, inferior; cent, central; pph, peripheral.

**Figure 1:**
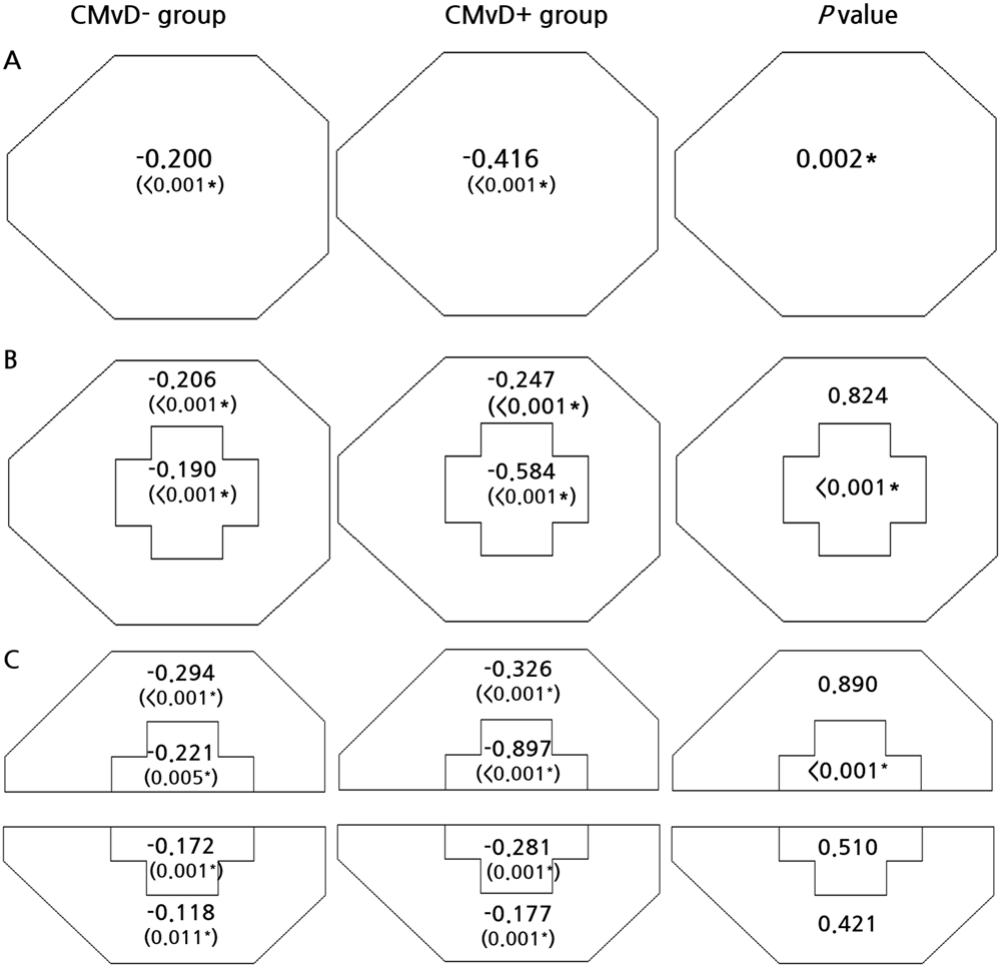
The global region (**A**), central 10° and peripheral 10° to 24° region (**B**) and superior and inferior regions of central 10° and peripheral 10° to 24° (**C**) in the choroidal microvasculature dropout (CMvD)− and CMvD+ groups, after adjusting for covariates. Significant rates of progression in these three maps were defined as negative slopes with P < 0.05, P < 0.025, and P < 0.0125, respectively. P value diagrams were evaluated using a linear mixed effects model with random effect variances, in which covariates were age, gender, spherical equivalent, central corneal thickness, mean follow-up intraocular pressure, visit-to-visit intraocular pressure fluctuation, and baseline mean deviation. In comparing two groups, a P value < 0.05 was considered statistically significant using a linear mixed effects model. Data are presented as means (dB/year). *Statistically significant using a linear mixed effects model.

The global rates of VF progression differed significantly in the eyes without CMvD (CMvD−) and with CMvD (CMvD+) (−0.200 dB/year vs. −0.416 dB/year; P = 0.002), after adjusting for covariates, including age, gender, SE, CCT, mean IOP, follow-up IOP fluctuation, and baseline MD (Fig. 1A). The rates of CVF progression in the central 10° and peripheral 10° to 24° regions differed significantly in the CMvD− and CMvD+ groups (−0.190 dB/year vs. −0.584 dB/year, P < 0.001), whereas the rates of VF progression in the peripheral VF region did not differ significantly in these groups (−0.206 dB/year vs. −0.247 dB/year, P = 0.824, Fig. 1B). When divided into superior and inferior regions (Fig. 1C), the rate of VF progression in the CMvD− and CMvD+ groups differed significantly in the superior center region (−0.221 dB/year vs. −0.897 dB/year, P < 0.001), but not in the inferior center region (−0.172 dB/year vs. −0.281 dB/year, P = 0.510).

Multivariate analysis using a linear mixed model that controlled for all covariates showed that the presence of CMvD (P < 0.001) and IOP fluctuation during follow-up (P = 0.025) were independent risk factor for global rapid rates of VF progression. Moreover, the presence of CMvD and IOP fluctuation during follow-up were independently associated with rapid VF progression rate in the superior center as well as in the center (P < 0.05 each). CCT was also independently associated with a rapid rate of VF progression in the superior central region (P = 0.009, Table 2).

**Table 2:** Clinical variables (P values) associated with rapid rates of global and regional visual field progression.

Clinical variables	Global	Central 10° and peripheral 10° to 24° map	Superior and inferior central and peripheral map
**Age**, **years**			
**Gender**			
**SE**, **D**			
Presence of CMvD	Global (0.001*)	Central (<0.001*)	Superior central (<0.001*)
CCT			Superior central (0.009*)
**Mean F/U IOP**			
F/U IOP fluctuation	Global (0.025*)	Central (0.02*)	Superior central (0.008*)
**Baseline MD**			

Blank cells indicate non-significant factors in the model. *Statistical significance in a linear mixed-effects model.

Abbreviations: D, diopter; F/U, follow-up; CCT, central corneal thickness; IOP, intraocular pressure; MD, mean deviation; CMvD, choroidal microvasculature dropout; dB, decibel.

Table 3 shows the results of univariate and multivariate logistic regression analyses assessing clinical variables associated with overall VF progression, with a generalized estimating equation (GEE) model used to adjust for potential inter-eye associations of the same patients. Multivariate logistic analyses showed that the presence of CMvD, DH, and large β-PPA/disc area were independently associated with VF progression (P < 0.05 each).

**Table 3:** Univariable and multivariable logistic regression analysis with generalized estimating equation to determine clinical variables associated with overall visual field progression.

POAG	Univariable			Multivariable		
	Exp(B)	95% CI	P value	Exp(B)	95% CI	P value
Age (year)	0.997	0.961 to 1.034	0.855			
Gender	0.583	0.232 to 1.466	0.251			
Baseline IOP	0.971	0.807 to 1.169	0.755			
Mean F/U IOP	1.020	0.755 to 1.379	0.896			
Peak F/U IOP	1.000	0.796 to 1.256	0.999			
F/U IOP fluctuation	0.924	0.691 to 1.235	0.835			
CCT (µm)	1.005	0.991 to 1.020	0.493			
CMvD	4.216	1.654 to 10.746	0.003*	3.573	1.023 to 12.482	0.046*
DH	12.442	3.704 to 41.799	<0.001*	15.176	3.708 to 62.107	<0.001*
Baseline MD	0.945	0.744 to 1.270	0.665			
SE	0.896	0.756 to 1.137	0.268			
β-PPA/disc area	1.507	1.218 to 1.864	<0.001*	1.428	1.116 to 1.829	0.005*
F/U period	1.201	1.018 to 0.994	0.136			

*P < 0.05 in univariable and multivariable logistic regression analysis with generalized estimating equation (GEE). A GEE model was used to adjust for potential inter-eye associations of the same patients.

Abbreviations: IOP, intraocular pressure; CCT, central corneal thickness; AL, axial length; CMvD, choroidal microvasculature dropout; DH, disc hemorrhage; MD, mean deviation; SE, spherical equivalent; β-PPA, β-zone parapapillary atrophy; F/U, follow-up.

Table 4 shows the results of structural progression rates and frequency of progression based on the GPA software provided by the Cirrus spectral-domain optical coherence tomography (SD-OCT). The average and superior quadrant RNFL progression rates were significantly faster in the CMvD+ group than in the CMvD− group. The average and inferior hemi-macular ganglion cell inner plexiform layer (GCIPL) progression rates were also significantly faster in the CMvD+ group than in the CMvD− group. The event-based progression analysis shows that eyes with CMvD had significantly greater frequency of structural progression using both RNFL and GCIPL parameters compared to those without CMvD.

**Table 4:** Comparison of parapapillary retinal nerve fiber layer (RNFL) and macular ganglion cell inner plexiform layer (GCIPL) thickness progression rates and frequency of progression based on the GPA software of eyes with open-angle glaucoma with (CMvD+) and without (CMvD−) CMvD.

GPA progression rate	CMvD(−), n = 30	CMvD(+), n = 33	P value
**RNFL GPA progression rate**, **µm/yr**			
Average	−0.39 ± 0.59	−0.80 ± 0.66	0.009*
Superior quadrant	−0.52 ± 1.02	−1.18 ± 1.11	0.020*
Inferior quadrant	−1.06 ± 1.19	−1.45 ± 1.26	0.219
RNFL progression, n (%)^†^	7 (23.3%)	22 (66.7%)	0.001*
**GCIPL progression rate**, **µm/yr**			
Average	−0.40 ± 0.38	−0.72 ± 0.51	0.009*
Superior hemifield	−0.38 ± 0.42	−0.59 ± 0.51	0.091
Inferior hemifield	−0.44 ± 0.49	−0.90 ± 0.89	0.016*
GCIPL progression, n (%)^†^	3 (10.7%)	16 (48.5%)	0.002*

Data are reported as mean ± standard deviation or n (%). *P < 0.05 by independent t-tests unless otherwise indicated. ^†^Chi-squared tests.

Abbreviations: CMvD, choroidal microvasculature dropout; n, number; RNFL, retinal nerve fiber layer; GCIPL, ganglion cell inner plexiform layer; GPA, guided progression analysis.

**Figure 2:**
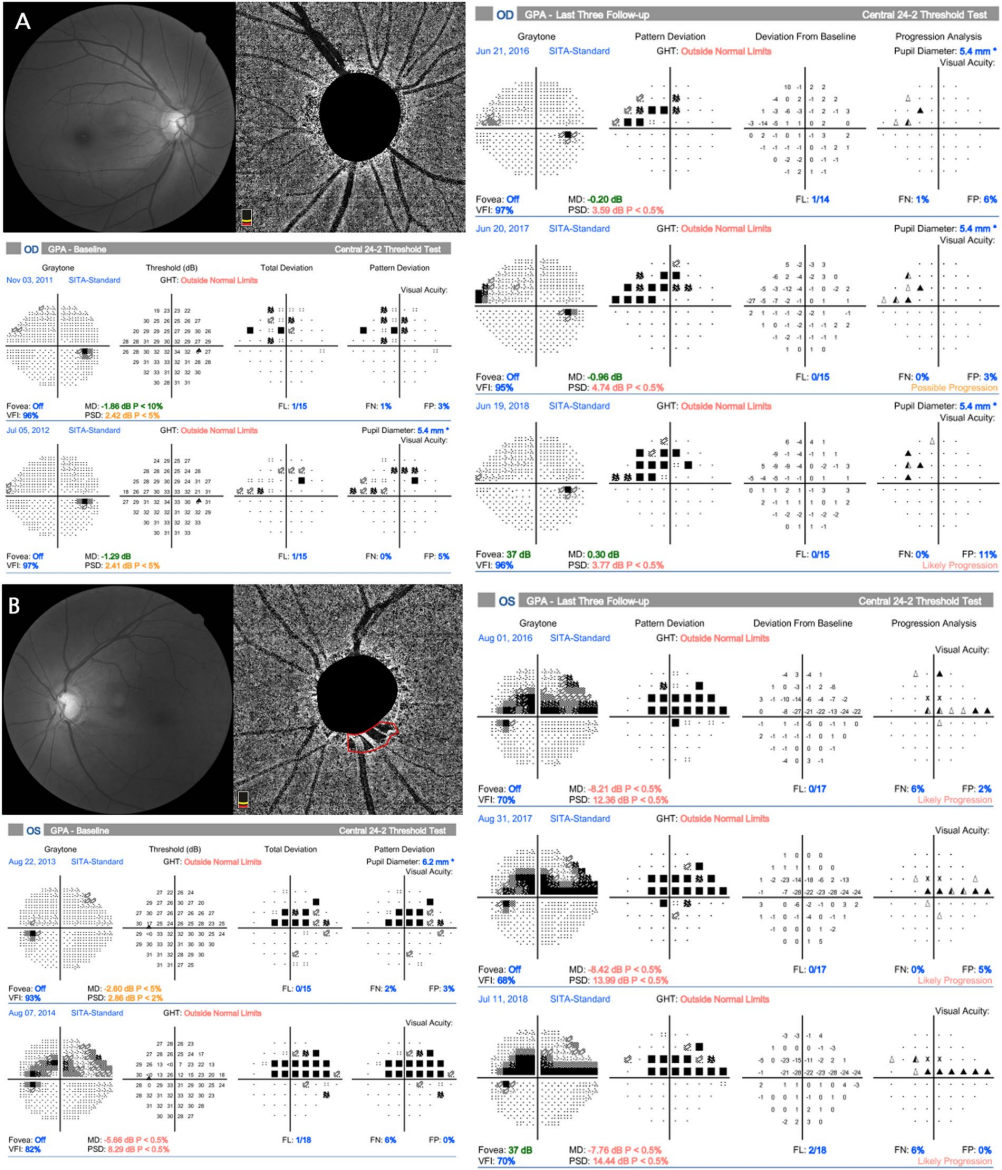
Representative cases showing the relationship between choroidal microvasculature dropout (CMvD) and central visual field (CVF) progression. (**A**) A 65-year-old woman with open-angle glaucoma (OAG) in the right eye and superior hemifield scotoma at baseline, who showed no significant visual field (VF) progression or CMvD during follow-up. (**B**) A 60-year-old woman with OAG in the left eye and superior hemifield scotoma, matched for age and VF severity with representative case (**A**) at baseline, who demonstrated rapid CVF progression, particularly in the superior center region, with a single inferior CMvD during follow-up. Red outline indicates the margin of the CMvD within β-zone parapapillary atrophy identified on the choroidal layer imaging of optical coherence tomography angiography.

Representative patients are described in Fig. 2A,B. A 65-year-old woman with OAG in the right eye but without CMvD showed superior hemifield scotoma at baseline, with no significant VF progression during follow-up. By comparison, a 60-year-old woman with OAG in the left eye and CMvD, matched for age and VF severity with representative case in Fig. 2A at baseline, showed a single inferior CMvD on the choroidal layer during OCT-A imaging and a rapid CVF progression rate, particularly in the superior central region.

## Discussion

CVF damage is common in eyes with early-stage OAG^12,13^, with rapid progression of CVF loss during the course of disease being devastating to glaucoma patients. The current study showed that CVF progression rates at the 12 central-most points on 24-2 VF tests were significantly higher in CMvD+ than in CMvD− eyes. This finding was further validated by the results of a multivariate linear mixed model, which showed that the presence of CMvD was independently associated with rapid CVF loss after controlling for other covariates related to VF progression. Consequently, along with DH and large β-PPA/disc area, detection of CMvD during follow-up was significantly associated with overall VF progression in eyes with OAG. These findings may help elucidate the nature of the relationship between the detection of CMvD during follow-up and the rate of CVF loss, as well as providing important insights into the clinical implications of CMvD on glaucoma prognosis related to CVF loss in eyes with early-stage OAG.

Choroidal vessel density (VD) maps of the ONH obtained by OCT-A show the presence of CMvD within the β-PPA. Although the pathogenesis of CMvD remains unclear, CMvD detected during follow-up is associated with progressive RNFL thinning or VF loss in patients with OAG^6,7^. Because CMvD represents a localized perfusion defect of choriocapillaries and choroidal microvessels^1,2,7^, the occurrence of CMvD may indicate hypo-perfusion to deep-layer structures of optic disc, such as prelaminar or laminar tissue. Because IOP-independent factors, such as vascular insufficiency to the ONH, may play an important role in the prognosis of OAG eyes^14–16^, CMvD may be considered a potential clinical sign or clue associated with glaucoma progression. Indeed, a higher percentage of VF progression was observed in eyes with than without CMvD, despite their having similar IOP profiles during follow-up. Nonetheless, further prospective studies are needed to determine whether baseline CMvD is associated with higher rates of VF progression.

CMvD during follow-up was recently reported to be associated with a greater likelihood of global VF progression in OAG patients^7^. Because glaucoma is a progressive disease, assessing the rate of VF progression, especially CVF progression, is undoubtedly important in patient management. Rapid CVF deterioration, even during early stages of glaucoma, can significantly hinder daily activities of glaucoma patients. The current study showed that global VF progression rates differed significantly in groups of patients with and without CMvD during follow-up (−0.416 dB/year vs. −0.200 dB/year, P = 0.002). More importantly, analyses of region-specific rates of VF progression showed a significantly faster progression rate of CVF in the CMvD+ than in the CMvD− group (−0.584 dB/year vs. −0.190 dB/year, P < 0.001), indicating the need for a more aggressive treatment strategy in patients with CMvD+ to hinder more rapid CVF loss during follow-up. By contrast, VF progression rates in the peripheral region (10° to 24°) did not differ significantly in these two groups (−0.247 dB/year and −0.206 dB/year, P = 0.824).

In addition to VF progression, the progression rate and progression incidence of RNFL and GCIPL were also compared between the 2 groups using trend- and event-based analysis. The CMvD+ group showed significantly faster average RNFL (−0.80 µm/year vs. −0.39 µm/year, P = 0.009) and GCIPL (−0.72 µm/year vs. −0.40 µm/year, P = 0.009) progression rates and higher incidence of structural progression compared to CMvD- group (P = 0.001 for RNFL, P = 0.002 for GCIPL, respectively). In line with faster superior CVF progression rate, the rate of GCIPL thickness loss was also significantly faster at the inferior hemi-macular region in the CMvD+ eyes compared to CMvD− eyes in our study (−0.90 µm/year vs. −0.44 µm/year, P = 0.016). Since the loss of GCIPL thickness is closely related to CVF defects^17,18^, the rapid loss of GCIPL thickness might have resulted in greater speed of CVF deterioration as seen in the CMvD+ eyes.

Several hypotheses may explain the faster rate of VF progression in the central 10° area of eyes with than without CMvD. Parafoveal scotoma in patients with early glaucoma is often associated with risk factors closely linked to vascular insufficiency to the ONH, such as hypotension, migraine, Raynaud’s phenomenon, and sleep apnea^19^. Further, patients with normal-tension glaucoma (NTG) accompanied by increased fluctuation of ocular perfusion pressure (OPP) due to excessive nocturnal BP dip often present with CVF scotoma^20^. This finding suggests that glaucoma pathogenesis in at least some OAG patients with CVF defects may involve a vascular mechanism. Consequently, eyes with signs of hypo-perfusion to the ONH, such as those with CMvD, may show a faster rate of VF progression in the CVF area than eyes without CMvD. Another explanation is that the most common pattern of VF progression in glaucoma is the deepening of an existing scotoma, followed by expansion^21,22^. Because CVF progression reflects the deepening or expansion of pre-existing initial CVF defects in eyes with CMvD, the rate of CVF progression is likely to be faster in eyes with than without CMvD.

The incidence of DH in the current study was 25% in the CMvD− and 29.5% in the CMvD+ group, respectively. The prevalence of DH in normal-tension glaucoma (NTG) patients is known to be around 25%^23^, which is consistent with that of our study consisting of NTG patients. It is well known that DH is significantly associated with future VF progression^15,24–26^, but there are conflicting results in the literature regarding the relationship between CMvD and DH. While recent study by Rao *et al*.^27^ showed that the prevalence of CMvD was significantly greater in OAG eyes with DH compared with those without DH, Suh *et al*.^1^ reported that DH was not found to be significantly associated with CMvD. In our study, there was no significant difference in terms of prevalence of DH between eyes with and those without CMvD, although the prevalence of DH in the CMvD+ group was slightly higher than that of CMvD-group. Prospective and longitudinal studies with large number of patients with DH will be needed in the future to elucidate this important relationship.

CMvDs are most frequently found in the inferotemporal (IT) sector within the β-PPA (7–8 o’clock), a location consistent with the macular vulnerability zone (MVZ) in the retina, which corresponds to the superior CVF area in 24-2 VF testing. This zone is particularly susceptible to glaucomatous damage^12^. Therefore, the detection of CMvD may be topographically related to poor prognosis of the superior CVF during the course of disease. In addition, VF defects progress more rapidly in the superior than in the inferior hemifield of eyes with OAG^28–30^. One study reported that the VF progression rate of the superior central 10° (−0.911 dB/year) in NTG eyes with initial superior hemifield defects was significantly faster than that of the inferior central 10° (−0.16 dB/year) in eyes with initial inferior hemifield defects^28^. Despite differences in the study populations and research designs, our results are in agreement with earlier findings, in that the VF progression rate was higher in eyes with CMvD in the superior (−0.897 dB/year) than in the inferior (−0.281 dB/year) 10° zone. All eyes (n = 88) in this study had NTG, a mean baseline IOP of 15.1 mmHg, and untreated IOPs within a normal range (<22 mmHg) during all outpatient visits.

Multivariate analysis using a linear mixed model showed that the detection of CMvD was an independent predictor of rapid VF progression in the center and superior center zones. Of the 44 eyes with CMvD in the present study, 42 eyes (95.5%) had CMvD in the IT sector within the β-PPA, whereas the other two eyes (4.5%) had CMvD in the superotemporal sector within the β-PPA. These findings were consistent with those of previous studies^2,31^. Although there is no clear explanation for the predominant occurrence of CMvD in the IT sector, the juxtapapillary choroid was found to be thinnest in the IT sector around the ONH^32,33^. Thus, the amount of blood flow from the branches of the SPC artery to the IT sector of the juxtapapillary choroid may be the lowest among various sectors around the ONH. This, in turn, may contribute to vascular insufficiencies, such as CMvD, in the IT sector of eyes with glaucoma.

Multivariate logistic regression analysis also showed that the detection of CMvD, DH, and the extent of β-PPA/disc were associated with overall VF progression in eyes with early-stage OAG. Although DH was shown to be a risk factor for glaucoma progression^34^, the presence of CMvD and β-PPA/disc area were also identified as risk factors for VF progression in the current study. Because the peripapillary choroidal microvasculatures are supplied by SPC arteries, which also perfuse prelaminar and laminar tissues^3,35^, the development of CMvD may indicate vascular compromise in the ONH. This may induce ischemic damage to the ONH by restricting the axonal transport of neurotrophic factors by mitochondria due to hypoxia and by releasing toxic substances that may also have negative effects on axonal function^36^. This, in turn, may facilitate the apoptosis of remaining RGCs and contribute to VF progression. In the current study, the β-PPA/disc area ratio was used to minimize the effect of photographic magnification error and to represent the dimensions of β-PPA^37,38^. β-PPA occurs more frequently in glaucomatous than in nonglaucomatous eyes and is associated with an increased risk of glaucoma progression^39–42^. Therefore, our findings are consistent with those of studies indicating that large β-PPA may be a risk factor for disease progression in OAG eyes.

This study had several limitations. First, because OCT-A is a relatively new technology, we acquired OCT-A images during follow-up after its introduction in 2016. To evaluate the temporal relationship between CMvD and CVF progression, prospective studies with OCT-A imaging acquired at baseline are needed. Despite this limitation, most of the CMvD+ eyes (64%, n = 28) presented with CVF defects, compared with only 18% (n = 8) of the CMvD− eyes, suggesting that a significant number of eyes with CVF defects may have had CMvD at baseline. In addition, because CMvD detected in OAG eyes during follow-up indicates a more rapid rate of CVF progression, these eyes deserve greater attention and more aggressive treatment during follow-up. Second, only early-stage OAG eyes were included. Thus, the results of this study may be difficult to apply to eyes with advanced glaucoma. However, we attempted to measure CVF progression rate in eyes with early-stage OAG, as the goal of glaucoma management is to detect and reduce the rate of glaucomatous VF progression at an early stage. In addition, a comparison of CVF progression rates in early-stage OAG eyes with and without CMvD may elucidate the pathogenic role of CMvD in OAG. Third, the retrospective nature of this study may have led to selection bias, as our patients were those examined at a university hospital, rather than being population-based. Thus, our patient cohort may not have had the same characteristics as similar patients in the general population. Fourth, CMvD was identified from en-face OCT-A images of the choroidal layer, a method with several limitations. For example, DH or large retinal vessels may project onto en-face choroidal layer images, inducing projection artifacts and rendering it difficult to detect or define CMvD boundaries. This study, however, included only eyes with CMvD clearly identified by two examiners. Fifth, we used Humphrey 24-2 VF than Humphrey 10-2 VF for the evaluation of CVF progression. Although 24-2 VF testing is routinely used in glaucoma patients, particularly at early stage of glaucoma, as in our study patients, it is not ideal for the detection of CVF progression due to its non-physiological test point distribution in the central 10° area^43^. In the current study, Humphrey 10-2 VF test might have increased retinal sensitivity to detect CVF defects as well as VF progression in the CVF area. Finally since our eyes represent Korean NTG eyes with and without CMvD referred to tertiary clinic, our data from a single ethnic group may not be generalized to other races or the general population. In the future, large-scale studies involving various races will be necessary.

In conclusion, this study showed significant regional differences in the rates of VF progression between OAG eyes with and without CMvD, with CMvD+ eyes showing significantly faster CVF progression rates than CMvD− eyes. Detection of CMvD during follow-up was found to be an independent predictor of rapid CVF progression, particularly in the superior center VF region, in eyes with early-stage OAG. Detection of CMvD during follow-up may be an important clinical marker for rapid CVF progression, requiring more aggressive management.

## Methods

### Study participants

The protocol of this retrospective study was approved by the Institutional Review Board of Asan Medical Center and conformed to the principles of the Declaration of Helsinki. The need for written informed consent was waived by our IRB due to its retrospective study design. The medical records of consecutive OAG patients who were examined between March 2011 and December 2013 at the glaucoma service of the Asan Medical Center, Seoul, Korea, were reviewed.

At the initial glaucoma work-up, each patient underwent a comprehensive ocular examination, including a review of past medical history, assessment of best-corrected visual acuity (BCVA) with refraction, slit-lamp biomicroscopy, Goldmann applanation tonometry, and gonioscopy. AL was determined by IOL master (Carl Zeiss Meditec, Dublin, CA, USA) and CCT by ultrasound pachymetry (DGH-550; DGH Technology, Inc., Exton, PA, USA). Patients also underwent dilated color fundus photography (Canon^®^, Tokyo, Japan), ONH stereoscopic photography, red-free RNFL photography (Canon^®^), Humphrey field analyzer Swedish Interactive Threshold Algorithm 24-2 VF testing (Carl Zeiss Meditec), and spectral-domain optical coherence tomography (SD-OCT, Carl Zeiss Meditec). A reliable VF assessment was defined as a VF test with a false-positive and -negative errors <15%, and a fixation loss <20%.

OAG was defined as the presence of an open iridocorneal angle; the appearance of glaucomatous ONH damage, consisting of neuroretinal rim thinning, notching, or RNFL defect; and a glaucomatous VF defect regardless of IOP level. A glaucomatous VF defect was defined according to the Anderson’s criteria^44^ and confirmed on two consecutive reliable VF tests. If the first VF test showed glaucomatous defects, the first result was excluded from analysis to obviate learning effects. The second VF was performed within 2–4 weeks after the first perimetry test. All OAG patients were followed up every 4–6 months by red-free fundus photography, ONH stereoscopic photography, VF, and SD-OCT. SBP and DBP were also measured during follow-up visits. MOPP was calculated as described^45^.

Patients were included if they (1) were ≥18 years-old at initial presentation, (2) had OAG with visible β-PPA on fundus photography, (3) had a BCVA ≥ 20/40 with a SE within ±6 diopters, (4) were followed up for a minimum of 4 years, with availability of at least 5 reliable VF datasets, (5) had a VF MD greater −6 dB at initial presentation, enabling assessment of VF progression rates of early-stage OAG eyes^44^, and (6) had undergone one or more OCT-A test during follow-up. The affected eye was selected in patients with unilateral disease. If both eyes of a patient had OAG and met the inclusion criteria, both eyes were included in the analyses.

Patients were excluded if they had severe myopic macular changes, including posterior staphyloma; a history of intraocular surgery; ocular diseases other than glaucoma, such as diabetic retinopathy; unreliable VF test results; cataract more than C2, N2, or P2 based on Lens Opacities Classification System III^46^; or a systemic disease that could influence the VF tests. Eyes were also excluded if they had undergone surgical or laser treatment for glaucoma during follow-up.

### Optical coherence tomography angiography

All subjects underwent OCT-A imaging with a commercially available OCT-A (AngioVue; Angio Disc mode, Optovue, Inc.), which uses a split-spectrum amplitude-decorrelation angiography algorithm to assess the dynamic motion of red blood cells, and presents a three-dimensional angiogram of perfused retinal vasculature^47^. The AngioVue provides vascular information at various user-defined retinal layers^48^.

The choroidal microvasculature in the parapapillary area was evaluated on choroidal layer. This layer is under the retinal pigment epithelium, including the choroid and sclera. CMvD within the β-PPA was defined as a complete loss of the choriocapillaries and microvasculature without any visible microvasculature network^1,31,45,49^. CMvD was identified when the minimum angular width was greater than 200 µm or that of the central retinal vein^1,7^. Two independent glaucoma specialists (Y.H.J. and J.K.) who were blinded to the patient’s clinical information reviewed all choroidal layer images and identified the presence of CMvD. Disagreements between the two observers were resolved by a third adjudicator (M.S.K.). Care was taken to avoid false-positive results due to shadowing by floaters or overlying retinal vessels. The control group consisted of OAG eyes without CMvD from the same database that met the inclusion criteria and were matched to eyes with CMvD by age (≤5 years) and VF MD (≤1 dB) at baseline.

### β-zone parapapillary atrophy and disc hemorrhage

The size of the β-PPA and the occurrence of DH were evaluated by the two glaucoma specialists (Y.H.J. and J.K.) using stereoscopic ONH photography. ${\rm{The}}$ β-PPA was defined as described^50,51^. The β-PPA and disc margin were manually demarcated, and their areas were measured using ImageJ software (version 1.52; Wayne Rasband, National Institutes of Health, Bethesda, MD, USA). The calculated β-PPA/disc area represented the size of the β-PPA independent of optic disc size while minimizing the effects of photographic expansion error^38^. The glaucomatous DH was defined as an isolated splinter- or flame-shaped hemorrhage on the optic disc or peripapillary area, extending to the disc border^45^. All DHs occurring during the follow-up period were recorded.

### Outpatient follow-up

After the diagnosis of OAG, all eyes were managed with (96.5%) or without (3.5%) topical IOP-lowering medications, at the discretion of the treating physician (M.S.K.), and/or confirmation of progression. At last follow-up, topical IOP-lowering medications included prostaglandin analogues in 81.8%, combinations of beta-blockers and carbonic anhydrase inhibitors in 50%, and brimonidine in 18.2%. Baseline IOP was measured before the treatment. The mean, peak, and fluctuation of IOP during follow-up period were calculated. VF progression was determined by Early Manifest Glaucoma Trial criteria^52–56^, using GPA. Only likely progression in the GPA software was considered VF progression in the current study.

### VF defects location and progression rates

To determine the location of VF defects and their progression rates, the central and peripheral VF regions were defined as described^32^. CVF was defined within 12 points of a central 10° of fixation, with the peripheral VF region defined as the area outside 10° of fixation. In addition, the central 10° and peripheral 10° to 24° maps were divided into superior and inferior areas to analyze hemifield regional progression rates. Two test locations within the blind spot were excluded. The rationale for separate analyses of superior and inferior hemifields was that VF progression in OAG eyes may show different progression rates depending on the location of initial hemifield VF defects^28,57^. A CVF defect at initial presentation was defined as a glaucomatous VF defect in one hemifield within 10° of fixation, with at least one point at P < 0.01 lying at the two innermost parafoveal points on the pattern deviation plot^19^, regardless of extension to 10° to 24° VF area. Eyes without CVF defects consisted of those with clusters only in the 10° to 24° regions of both hemifields.

The average VF mean sensitivity (MS) in each region was used to calculate the progression rate in that region, as described^58^. All individual values at each test location of the total deviation plot within the global, central 10°, peripheral 10° to 24°, superior and inferior central 10°, and superior and inferior peripheral 10° to 24° regions were averaged to yield the average MS for each follow-up^32^. In averaging individual values, VF MS (in dB scale) at each test point was converted to the linear scale of 1/Lambert (1/L), which was averaged to obtain mean MS values in each region^59,60^. VF progression rates were calculated as the changes in VF MS from baseline of each area of the same eye.

### RNFL and GCIPL progression analysis

To determine the incidence and rate of structural progression, trend- and event-based analyses were performed for the parapapillary RNFL and macular GCIPL thickness parameters (at global, superior, and inferior regions) using Cirrus SD-OCT data (Cal Zeiss Meditec, Inc., Dublin, CA). The rates of structural progression were provided by the GPA software using linear regression analysis (expressed in µm/year). For the event-based analysis, only “likely progression” in the GPA software was regarded as structural progression in the current study. Images with artifact or a signal strength <6 and cases of segmentation failure were excluded from the linear regression analysis.

### Statistical analyses

Inter-examiner agreements regarding the presence of CMvD and β-PPA/disc area were assessed using Kappa statistics and ICCs. Categorical were compared in the CMvD+ and CMvD− groups by chi-squared tests and continuous variables were compared by independent t-tests.

To calculate rates of VF progression for each region, a linear mixed model was used to account for the correlated nature of the outcomes within each individual eye^57^. Models were fitted with fixed coefficients (fixed effects) of follow-up time, patient age, SE, CCT, mean follow-up IOP, follow-up IOP fluctuation, and baseline MD, with random intercepts and coefficients (random effects) of patient and eye (each eye nested within subject) representing the effect of time. VF progression rates for each region were compared in the CMvD+ and CMvD− groups using a linear mixed model. The probability levels considered statistically significant were P < 0.0125 for comparisons of the superior and inferior central 10° and peripheral 10–24° zones, P < 0.025 for comparison of the central 10° and peripheral 10–24° zones, and P < 0.05 for comparisons of the global 24-2 area^58,61^. Multivariate analysis using linear mixed models was used to determine the variables that influenced the VF progression rate in global and regional clusters; these analyses included as factors age, gender, SE, CCT, presence of CMvD, follow-up mean and fluctuation IOP, and baseline MD. Finally, univariate and multivariate logistic regression analyses with a GEE were used to determine the clinical factors associated with VF progression as defined by GPA, adjusting for potential inter-eye associations. Finally, to compare the rates of parapapillary RNFL and macular GCIPL thinning and incidence of progression based on the GPA software between the 2 groups, the independent t-test and chi-squared test were used respectively. Two-tailed P values < 0.05 were considered statistically significant. All statistical analyses were performed with R (version 3.5.1, R Foundation for Statistical Computing, Vienna, Austria) and SPSS (version 18.0, SPSS, Inc., Chicago, IL, USA) software.
